# Supplementary material for: Lineage-based scaling of germline intercellular bridges during oogenesis
Source: Development. 2024 Aug 27;151(16):dev202676. doi: 10.1242/dev.202676 (PMC11385318; doi:10.1242/dev.202676)
Supplement: Supplementary information [file develop-151-202676-s1.pdf]

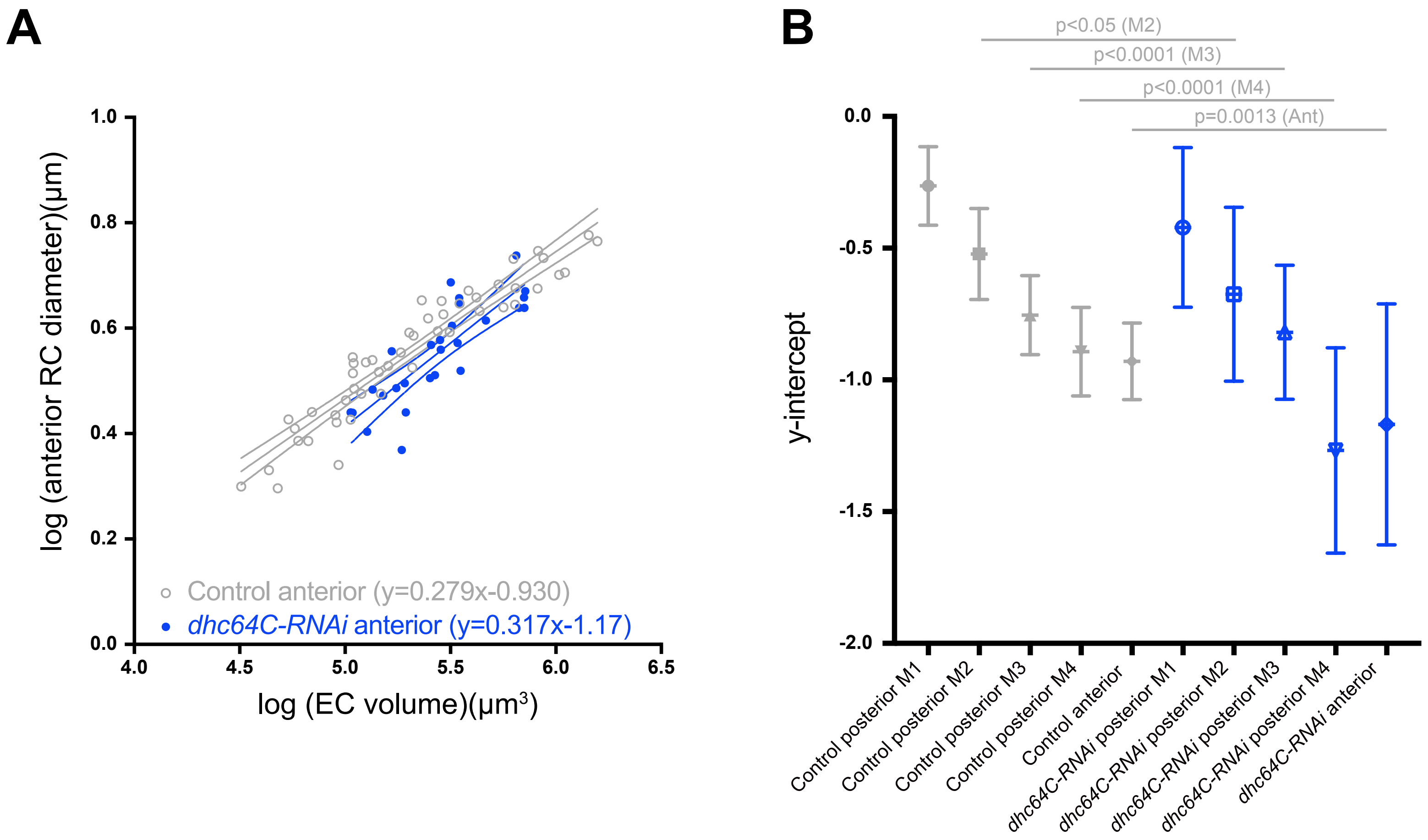

Fig. S1. Reducing oocyte growth has a modest effect on ring canal size and scaling. (A) log-log plot of egg chamber (EC) volume and anterior ring canal (RC) diameter from stage 5-10b. 95% confidence intervals are shown. (B) y-intercepts of regression lines from log-log plots of egg chamber volume and ring canal diameter (related to Fig. 2). Error bars are 95% confidence intervals. n=49 egg chambers for control and 28 egg chambers for *dhc64C-RNAi*.

Development • Supplementary information

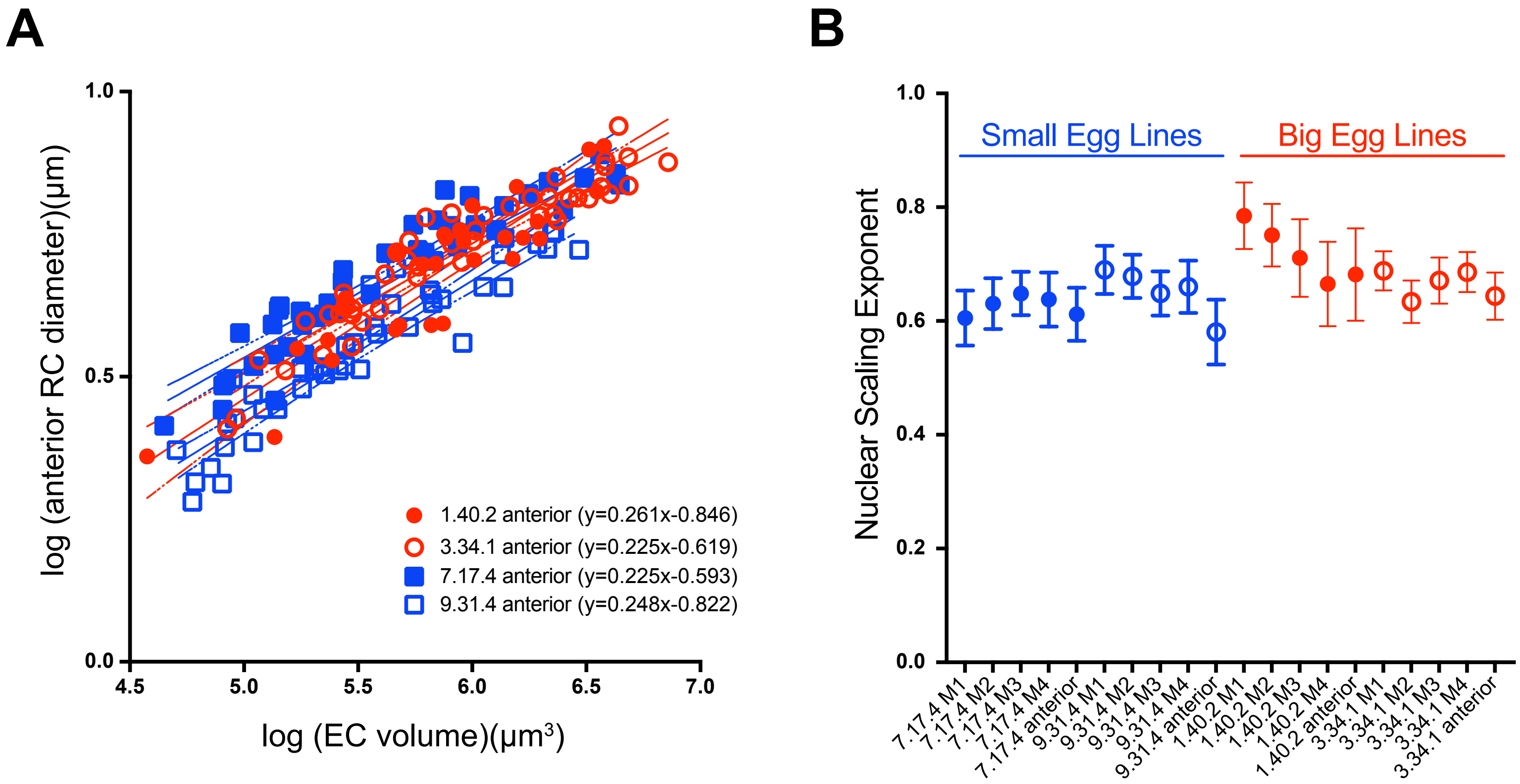

Fig. S2. Anterior ring canal and nuclear scaling in "big egg" and "small egg" *D. melanogaster* lines. (A) log-log plot of egg chamber (EC) volume and anterior ring canal (RC) diameter in two big egg (1.40.2 and 3.34.1) and two small egg (7.17.4 and 9.31.4) lines. 95% confidence intervals are shown. (B) Scaling exponent (+/- 95% confidence interval) for nuclei based on lineage. n=29 egg chambers for 1.40.2, 46 egg chambers for 3.34.1, 44 egg chambers for 7.17.4, and 43 egg chambers for 9.31.4.
