# Supplementary material for: Lineage-based scaling of germline intercellular bridges during oogenesis
Source: Development. 2024 Aug 27;151(16):dev202676. doi: 10.1242/dev.202676 (PMC11385318; doi:10.1242/dev.202676)
Supplement: Supplementary file 1 [file develop-151-202676_review_history.pdf]

Lineage-based scaling of germline intercellular bridges during oogenesis

Umayr Shaikh, Kathleen Sherlock, Julia Wilson, William Gilliland and Lindsay Lewellyn
DOI: 10.1242/dev.202676

Editor: Swathi Arur

Review timeline

Submission to Review Commons:	22 August 2023
First Submission to Development	3 January 2024
First Editorial decision:	8 January 2024
First Resubmission	19 April 2024
Second Editorial decision:	10 June 2024
Second Resubmission:	16 July 2024
Accepted:	19 July 2024

Reviewer 1

Evidence, reproducibility and clarity

The relationship between nuclear size and cell size has received a lot of attention. Less well-recognized is the problem of how other structures within the cell scale with size. This is a good question and the authors have a nice system - Drosophila female germline cysts - in which to study it. Here the authors show that lineage impacts ring canal scaling. This is an interesting finding that makes for neat biology; a simple way to think about it is that older ring canals have more time to mature (grow) than younger ones. The manuscript is beautifully and carefully written! It was fun to read. The experiments are straightforward, performed to a high standard, and generally well-presented.

Comments:

One way to think about the dhc-64C experiments presented in Figure 2 is that they are meant to test the hypothesis that ring canal size impacts scaling in such a way that transport across the four ring canals tends towards equilibrium over time. One possibility would therefore be that ring canals aren't programmed to grow to a particular final size but rather they grow at different rates until their diameters are the same. This seems to me an important distinction. It might be made by analysis of the arpC2-RNAi cells, since those ring canals are meant to be initially larger. Unfortunately I can't see the answer. The authors write that arpC2-RNAi "ring canals tended to be larger than those in similarly-sized control egg chambers," but that conclusion isn't obvious to me from the data in Figure 3B. The only difference I can see is that the M4 ring canals look to be consistently smaller in the experimental versus control egg chambers, especially at the final timepoint. Related to this concern, the authors write that "there was a consistent, but not significant decrease in the scaling exponents for the arpC2-RNAi egg chambers compared to controls," but I don't see this in the M1 (identical) or M2 (almost the same) ring canals. The scaling decrease is most pronounced at M4. All of the other ring canals seem to reach a final size that's equivalent to controls. What does this tell us about scaling? Is the M4 more sensitive to the effect of arpC2-RNAi? I note and appreciate that the data for M4 show a wide distribution and might have been impacted by outliers, which could be discussed.

© 2024. Published by The Company of Biologists under the terms of the Creative Commons Attribution License (https://creativecommons.org/licenses/by/4.0/).

The possibility that ring canal scaling "could generate eggs of different sizes" could use some elaboration (at least) as it does not seem to be especially well supported:

- Only one of the small egg lines had lower scaling exponents than the big egg lines, and it's a struggle for me to understand the extent of that difference based on the data shown. (Is it significant?).
- The authors conclude that "the effect of lineage on ring canal scaling is conserved, and it suggests that at least in one line, reducing posterior ring canal scaling could provide a mechanism to produce a smaller mature egg." The first part of this sentence is confusing for me since I don't know what is meant here by "conserved." The second part of the sentence is technically correct but disguises what I would consider the more meaningful and exciting finding. The 9.31.4 line produces the smallest eggs but does not demonstrate scaling differences in comparison to the big egg lines examined (91.40.1 and 3.34.1). The authors have therefore avoided/solved a "chicken and egg" ("fruit fly and egg"?) problem by showing that scaling and egg size can be decoupled!
- This point is not made very clearly in the discussion, which concludes with the suggestion that scaling could help explain why some insects produce much larger or much smaller eggs that fruit flies. I can only understand this to be the case if - as the authors point out - scaling "affect<s> the directed transfer of materials into the oocyte." That argument seems predicated on the possibility that these insects make the same amount of initial material then regulate how much is transferred. Seems like a costly way to go about it.

Minor comments:

I really had to look very closely to distinguish the little blue boxes from the little blue circles in panels 2C and especially 2D. I suggest using a different color instead of a different shape, or maybe splitting the graphs up.

The introductory material and the title of the paper emphasize the ring canal scaling question. This problem is somewhat obscured in the text by the side problem of nuclear scaling, which comes up frequently even though the results are not as thoroughly explored. Could the authors think about moving these data into a different, single figure for the sake of coherence? This is not a required revision. Just a thought.

I have two trivial comments regarding sentence structure in the text:
"Depletion of the linker protein, Short stop (Shot), or dynein heavy chain (Dhc64C), significantly reduced the biased transport at the posterior, which reduced oocyte size (Lu et al., 2021)." I suggest this sentence might be clearer if it was rewritten as "Depletion of either dynein heavy chain (Dhc64C) or the linker protein Short stop (Shot) significantly reduces biased transport at the posterior, in turn reducing oocyte size (Lu et al., 2021).

"Because nuclear growth has been shown to be tightly coupled to cell growth (Diegmiller et al., 2021), we can use nuclear size as a proxy for nurse cell size." I think it would help the reader to know that the Diegmiller study was performed using germline cysts in the Drosophila ovary; I paused when I got to this sentence because I initially read it as overly broad. I suggest "Recent work in demonstrates that nuclear growth is tightly coupled to cell growth in this system (Diegmiller et al., 2021), and we can therefore use nuclear size as a proxy for nurse cell size" or similar. This is certainly not a required revision, just a suggestion.

Significance

The manuscript is beautifully and carefully written! It was fun to read. The experiments are straightforward, performed to a high standard, and generally well-presented. The problem it addresses is an important/useful complement to other studies on the relationship between nuclear size and cell size. The paper identifies and characterizes differential scaling of ring canals, raising exciting mechanistic questions that can be addressed in future studies. I think it will be of interest to an audience cell and developmental biologists.

Reviewer 2

© 2024. Published by The Company of Biologists under the terms of the Creative Commons Attribution License (https://creativecommons.org/licenses/by/4.0/).

Evidence, reproducibility and clarity

In this manuscript, Umayr Shaikh and colleagues study the evolution of the size of subcellular structures during tissue growth. To do this, the authors use the Drosophila egg chamber as a model system, studying the growth rate of the intercellular bridges, also known as ring canals, connecting the oocyte to the nurse cells and the growth rate of the nurse cell nuclei during the development of the egg chamber. In particular, they focused their study on the ring canals and the nuclei of the 4 nurse cells, both of which are in direct contact with the oocyte but have a different lineage history. They show that first born ring canal ring grow more slowly than smaller ring canal that are the result of subsequent mitotic divisions. This scaling process is maintained when polarised transport between the nurse cells and the oocyte is reduced by decreasing the level of dynein. They demonstrate that manipulation of the size of the ring canals by arpC2 RNAi does not radically alter scaling. Furthermore, by inactivating an uncharacterised gene CG34200, they show that additional mitotic division does not affect the scaling of the annular canal and nucleus.

Significance

Major comment

These results are based on new and original observations. The results are clear and well documented. However, this work is very descriptive in its current state and in the absence of a mechanism for this lineage-based scaling process.

I am surprised that the author has not considered controlling the impact of cell cycle regulation on this scaling process, especially as the work of Dorherty et al. has shown that this type of regulation is essential for regulating the size of nurse cell nuclei. The authors should test the impact of at least dacapo and cyclin E in this process.

Without a mechanism for the scaling process, this manuscript is more suitable for a specialize journal

<u>Reviewer 3</u>

Evidence, reproducibility and clarity

Coordinating subcellular size of organelles is important for normal cell function. This research investigates how the size of intercellular bridges called ring canals in Drosophila egg chambers is regulated. The work builds on previous research in Change Tan's lab that reported ring canal size varies by lineage - the ring canal from the first germ cell mitotic division (M1) is larger than those resulting in subsequent divisions during egg chamber formation. Shaikh et al. probe this linkage between lineage and ring canal size during egg chamber development by testing the relationship with flow through the ring canals, initial ring canal size, and number of mitotic divisions. They also investigate whether ring canal size per se affects egg size. The quantification is carefully done.

The results strongly reinforce the overall conclusion that lineage is the main driver of ring canal size. They report that growth of younger ring canals is slightly faster than old ones, suggesting a "catch-up" mechanism. However, neither the flow through ring canals nor their initial size has an apparent effect on the relationship with lineage.

General comments:

The authors derive scaling relationships between nurse cell or ring canal size and egg chamber volume to address their hypotheses. The most interesting observation is the relatively accelerated growth of young versus old ring canals attaching the oocyte to nurse cells, each from a different mitosis. Another perhaps simpler way to do this is to determine the ratios of the diameters of M1 to M4 ring canals as egg chambers develop. Does the ratio stay the same?

Specific comments:

© 2024. Published by The Company of Biologists under the terms of the Creative Commons Attribution License (https://creativecommons.org/licenses/by/4.0/).

Fig. 1:

1. For those who forgot their algebra, it would be helpful to explain that the log-log plots were used to derive a line equation (y=mx + b) and why that is useful in this context. In the case of a log-log plot, what does the y-intercept mean biologically? Is it simply a way to compare two things or does it indicate real measurements such as volume or ring canal size? Also, the slope of the line is being used as a scaling value. Be careful to define the terms "scaling" and "scaling exponent".
2. Are four significant digits called for in calculating slope? The figure has 4 significant digits, the text has three.
3. Why is isometric scaling 0.66 - is that microns squared over microns cubed? Please explain.
4. Were all four posterior nuclei measured? The figure indicates just M1 and M4. It is hard to explain why all four posterior nuclei are bigger than anterior when one of the four is the same age as the anterior nucleus.
5. In panel D, a conclusion is, "Further, the scaling exponent [slope] for the anterior ring canals, which are also formed during the fourth mitotic division, was not significantly different from that of the posterior M4 ring canals". Anterior is 0.23, M4 is 0.25. These seem different to me. How is significance determined? Were any of the scaling exponents in M1, M2, M3, M4 or Anterior significantly different?

Fig. 2: Less flow through M4 drives faster RC growth? No.

6. References are needed for the statements about biased transport to the oocyte.
7. In panel C, why are the scaling exponents (slopes) of the controls bigger than in Figure 1B? The controls look hyper allometric in Fig. 2.
8. In panel D it is impossible to pick out the control posterior vs anterior lines - use different colors as in Figure 1. Why do the control lines for posterior and anterior merge?
9. In pane E, it would be helpful to plot the y-intercepts separately, too.

Fig. 3: increasing initial RC size does not dramatically alter scaling.

10. Scaling of what? RC size?
11. Since there was no effect, "dramatically" should be deleted from the section title.
12. Clarify this sentence: If ring canal size inversely correlates with scaling, then increasing initial ring canal diameter should reduce the scaling exponent.
13. I cannot see differences in RC size in the panel A images. More importantly, this method altering ring canal size is limited. A more direct way is overexpression of HtsRC (https://doi.org/10.1534/genetics.120.303629).
14. How does panel B show that RCs are larger in arpC2 KD? Fig. S1A has smaller y-intercept for control. Again, it is impossible to see which lines go with which M and which genotype.
15. Panel E is interesting and would fit better in Figure 1.

Fig. 4: Additional mitotic division doesn't affect RC or nuclear scaling.

16. Panels D & G are clear - should include significance indications.

Fig. 5: small and big lines

17. The conclusion from panels B and C that reducing RC scaling could lead to smaller mature eggs is a stretch. Without looking at the rest of the lines these data are preliminary and detract from the rest of the paper.

Significance

Overall, this work is an extension and reinforcement of information previously available rather than providing significant new insight. Researchers in Drosophila oogenesis will be interested.

Manuscript number: RC-2023-02139
Corresponding author(s): Lindsay, Lewellyn

1. General Statements [optional]

The size of subcellular structures must be tightly controlled to maintain normal cell function; this is especially important when cells are part of developing tissues or organs. Despite

© 2024. Published by The Company of Biologists under the terms of the Creative Commons Attribution License (https://creativecommons.org/licenses/by/4.0/).

its importance, few studies have determined how the size of organelles or other structures is maintained during development, when cells are growing, dividing, and rearranging. We have chosen to use the fruit fly egg chamber as a model system to determine the size scaling of an essential structure, the germline intercellular bridge, or ring canal. Similar intercellular bridges are found in the developing gametes of organisms from fruit flies to humans, but to our knowledge, an analysis of the scaling of these structures during development has not been performed. Within the egg chamber, there are 15 ring canals that connect the nurse cells to each other and to the developing oocyte; these ring canals are formed following each of the four germ cell divisions that produce the germ cell cyst. Because of the stereotypical arrangement of cells within the cluster, it provided us with the opportunity to determine whether ring canal scaling differed based on position within the germ cell cluster and/or lineage. Recent work has demonstrated that in this system, there is a hierarchy to nurse cell size, with the largest nurse cells at the posterior and the smallest nurse cells at the anterior. We initially hypothesized that ring canal size and growth would follow that of the nurse cells; the largest, fastest growing ring canals would be found at the posterior, connected directly to the oocyte, and the smallest, slowest growing ring canals would be found at the anterior. We were surprised to instead find that ring canal scaling was more closely related to lineage than to position within the germ cell cluster; specifically, ring canals formed following the first mitotic division were the largest and increased in size more slowly than the smaller ring canals that were formed during the subsequent mitotic divisions. Reducing directed transport through the ring canals, increasing initial ring canal size, or inducing a 5th germ cell division did not disrupt this pattern of ring canal scaling. Further, analysis of a set of lines that produce eggs of different sizes revealed that altering ring canal size or scaling could provide a mechanism to alter final egg size. Our initial results suggest that ring canal size could have a modest impact on ring canal scaling, and we hope that with the planned experiments we will be able to either confirm this contribution or rule it out. Interestingly, our analysis also revealed that the nurse cell nuclei exhibit scaling differences based on lineage. The nuclei within the nurse cells formed during the first mitotic division tended to be smaller but increased in size faster compared to the nuclei within nurse cells formed during the later mitotic divisions. This work has laid the foundation for future studies to determine the molecular mechanisms that establish and maintain these scaling relationships during tissue growth.

We would like to thank the reviewers for their careful reading of the manuscript and thoughtful recommendations. The suggested changes have significantly improved the manuscript, and after the planned revisions, we believe that our data will provide important insight into the size scaling of a conserved structural feature found in the developing sperm and eggs of organisms throughout the animal kingdom, therefore making it of interest to a broad developmental biology audience.

2. Description of the planned revisions

Planned Revisions based on comments from Reviewer #1

- The introductory material and the title of the paper emphasize the ring canal scaling question. This problem is somewhat obscured in the text by the side problem of nuclear scaling, which comes up frequently even though the results are not as thoroughly explored. Could the authors think about moving these data into a different, single figure for the sake of coherence? This is not a required revision. Just a thought.
    - *We have moved the nuclear scaling data from Fig. 5 into Fig. S3, and once we have analyzed the data from the planned experiments (over-expressing either HtsRC or the active form of myosin), then we will have a better idea of whether we should move the rest of the nuclear scaling data out of the main part of the paper, consolidate it into a single figure (as Reviewer #1 suggests), or keep some of it in the main figures.*

Planned Revisions based on comments from Reviewer #3

- I cannot see differences in RC size in the panel A images. More importantly, this method altering ring canal size is limited. A more direct way is overexpression of HtsRC (https://doi.org/10.1534/genetics.120.303629).
    - *We have requested and just recently received the line to over-express HtsRC in the germline. We plan to cross this UAS line to the mat□Tub-GAL4 which expresses GAL4 beginning around stage 3 of oogenesis. Because crossing this UAS*

© 2024. Published by The Company of Biologists under the terms of the Creative Commons Attribution License (https://creativecommons.org/licenses/by/4.0/).

line with this GAL4 line produced egg chambers with larger ring canals in the original study[2]*, we do not anticipate any technical issues with this experiment. We will incorporate the results from analysis of these egg chambers in the revised manuscript.*

o *To further explore the effect of ring canal size on scaling, we will also be testing a condition that we hope will have the opposite effect on ring canal size; expression of a phosphomimetic version of the non-muscle myosin II regulatory light chain, encoded by spaghetti squash (Sqh)(UAS-sqh^{E20E21}). We plan to cross this UAS line to two different GAL4 drivers (nos-GAL4, which expresses GAL4 in a pulse during early oogenesis and then in another pulse in mid-oogenesis and the mat□Tub-GAL4 which expresses GAL4 beginning around stage 3 of oogenesis). We know that expression of sqh^{E20E21}will reduce the size of the ring canals that connect the nurse cells to each other, but it is possible that the posterior ring canals will not show a strong phenotype. In a study that looked at egg chambers homozygous for a mutation in the myosin binding subunit of the myosin phosphatase, DMYPT, which should also increase sqh phosphorylation, it was shown that the posterior ring canals were larger than those connecting nurse cells* [1]*. Therefore, it is possible that this condition may not allow us to consistently reduce the size of all ring canal types; however, if we do see a* significant reduction in posterior ring canal size in these egg chambers, we will include these data in the revised manuscript.

- In panel 2E, it would be helpful to plot the y-intercepts separately, too.
  - *Based on the analysis of the data from the proposed experiments, we will consider plotting the y-intercepts separately for the various conditions.*

3. Description of the revisions that have already been incorporated in the transferred manuscript

Revisions made based on comments from Reviewer #1

- One way to think about the dhc-64C experiments presented in Figure 2 is that they are meant to test the hypothesis that ring canal size impacts scaling in such a way that transport across the four ring canals tends towards equilibrium over time. One possibility would therefore be that ring canals aren't programmed to grow to a particular final size but rather they grow at different rates until their diameters are the same. This seems to me an important distinction. It might be made by analysis of the arpC2-RNAi cells, since those ring canals are meant to be initially larger. Unfortunately, I can't see the answer.
  - *Reviewer #3 suggested determining the ratio of the diameter of the M1 ring canal to the M4 ring canal. If ring canals grow toward equilibrium (to achieve a similar final size), then we would expect to see this ratio approach 1; when we performed this analysis, we saw that the ratio did decrease as the egg chambers increased in volume, but it never quite reaches a ratio of 1. We have added a supplemental figure (Fig. S1) showing these data and incorporated this idea into the text within the results and discussion sections.*
  - *Although it would be informative to determine whether ring canals that all started with a similar diameter would grow at the same rate, we have not found a condition that would provide the opportunity to test this hypothesis. We hope that the planned experiments will provide us with a way to test this hypothesis; we will determine the M1/M4 ratio in egg chambers over-expressing either HtsRC or sqh^{E20E21} and see whether this ratio still decreases as egg chamber volume increases.*
  - *Once we perform the planned experiments to either increase or decrease ring canal size, then we can determine whether we need to further modify Fig. 3 to highlight these size differences between ring canals in the arpC2-RNAi egg chambers or whether we will instead focus more on the results of the planned experiments.*
- The authors write that arpC2-RNAi "ring canals tended to be larger than those in similarly-sized control egg chambers," but that conclusion isn't obvious to me from the

© 2024. Published by The Company of Biologists under the terms of the Creative Commons Attribution License (https://creativecommons.org/licenses/by/4.0/).

data in Figure 3B. The only difference I can see is that the M4 ring canals look to be consistently smaller in the experimental versus control egg chambers, especially at the final timepoint.

  - *To further clarify the difference in ring canal size between the control and the arpC2-RNAi egg chambers, we have added additional explanation to the results section to highlight that the y-intercepts of the lines of best fit are significantly higher in the arpC2-RNAi egg chambers at each stage. This demonstrates that given an egg chamber volume, the ring canals will be larger in egg chambers depleted of ArpC2 than in the controls.*

- The authors write that "there was a consistent, but not significant decrease in the scaling exponents for the arpC2-RNAi egg chambers compared to controls," but I don't see this in the M1 (identical) or M2 (almost the same) ring canals. The scaling decrease is most pronounced at M4. All the other ring canals seem to reach a final size that's equivalent to controls. What does this tell us about scaling? Is the M4 more sensitive to the effect of arpC2-RNAi? I note and appreciate that the data for M4 show a wide distribution and might have been impacted by outliers, which could be discussed.
  - *We have separated the arpC2-RNAI ring canal scaling data by lineage (Fig. S2), and we have color-coded the data in Fig. 3B (as suggested by Reviewer #3).*
  - *We have expanded the discussion of these results and their implications, and we have added a line in the results section to address this wide distribution of the M4 ring canal sizes.*
- The possibility that ring canal scaling "could generate eggs of different sizes" could use some elaboration (at least) as it does not seem to be especially well supported:
  - Only one of the small egg lines had lower scaling exponents than the big egg lines, and it's a struggle for me to understand the extent of that difference based on the data shown. (Is it significant?).
    - *We have restructured this section of the results and modified Fig. 5 to highlight similarities and differences between the four lines. In the results section (and in the figure legend), we have stated that when we compared the slopes of the regression lines for all four lines, there was a significant difference for M1, M2, and M4 (Fig. 5C, D, and F). We have also modified the results section to highlight that although the slopes for line 9.31.4 was not different from the two big egg lines, the intercepts were significantly different for M1, M2, M3, and M4 ring canals. We moved the nuclear scaling data to Fig. S3 to simplify the figure.*
  - The authors conclude that "the effect of lineage on ring canal scaling is conserved, and it suggests that at least in one line, reducing posterior ring canal scaling could provide a mechanism to produce a smaller mature egg." The first part of this sentence is confusing for me since I don't know what is meant here by "conserved." The second part of the sentence is technically correct but disguises what I would consider the more meaningful and exciting finding. The 9.31.4 line produces the smallest eggs but does not demonstrate scaling differences in comparison to the big egg lines examined (1.40.1 and 3.34.1). The authors have therefore avoided/solved a "chicken and egg" ("fruit fly and egg"?) problem by showing that scaling and egg size can be decoupled!
    - *We have modified the first part of the sentence to clarify our point. We appreciate this suggestion and have modified the text in the results section to further elaborate on the results.*
  - This point is not made very clearly in the discussion, which concludes with the suggestion that scaling could help explain why some insects produce much larger or much smaller eggs that fruit flies. I can only understand this to be the case if - as the authors point out - scaling "affect<s> the directed transfer of materials into the oocyte." That argument seems predicated on the possibility that these insects make the same amount of initial material then regulate how much is transferred. Seems like a costly way to go about it.
    - *We have modified this section of the discussion.*
- I really had to look very closely to distinguish the little blue boxes from the little blue circles in panels 2C and especially 2D. I suggest using a different color instead

© 2024. Published by The Company of Biologists under the terms of the Creative Commons Attribution License (https://creativecommons.org/licenses/by/4.0/).

of a different shape, or maybe splitting the graphs up.
    - *We have made the shapes larger in Fig. 2C (nuclear sizes), and we have split the ring canal size data into Fig. 2D, E and made the shapes larger. The legend has been modified to reflect this change.*
- "Depletion of the linker protein, Short stop (Shot), or dynein heavy chain (Dhc64C), significantly reduced the biased transport at the posterior, which reduced oocyte size (Lu et al., 2021)." I suggest this sentence might be clearer if it was rewritten as "Depletion of either dynein heavy chain (Dhc64C) or the linker protein Short stop (Shot) significantly reduces biased transport at the posterior, in turn reducing oocyte size (Lu et al., 2021).
    - *We have made this change.*
- "Because nuclear growth has been shown to be tightly coupled to cell growth (Diegmiller et al., 2021), we can use nuclear size as a proxy for nurse cell size." I think it would help the reader to know that the Diegmiller study was performed using germline cysts in the Drosophila ovary; I paused when I got to this sentence because I initially read it as overly broad. I suggest "Recent work in demonstrates that nuclear growth is tightly coupled to cell growth in this system (Diegmiller et al., 2021), and we can therefore use nuclear size as a proxy for nurse cell size" or similar. This is certainly not a required revision, just a suggestion.
    - *We have made this change.*

<u>*Planned Revisions based on comments from Reviewer #3*</u>

- Reviewer #3 asked: Does the ratio of the diameter of M1 to M4 stay the same?
    - *We have performed this analysis in the control egg chambers (from Fig. 1), and we found that the ratio does not stay the same, but that it tends to decrease as the egg chamber increases in volume. We plotted the log of egg chamber volume versus this ratio, and the equation for the regression line was y = -0.166x + 2.32, which was significantly different from a slope of 0 (included in Fig. S1).*
- It would be helpful to explain that the log-log plots were used to derive a line equation (y=mx + b) and why that is useful in this context. In the case of a log-log plot, what does the y-intercept mean biologically? Is it simply a way to compare two things or does it indicate real measurements such as volume or ring canal size? Also, the slope of the line is being used as a scaling value. Be careful to define the terms "scaling" and "scaling exponent".
    - *We have added additional explanation in the results section.*
- Are four significant digits called for in calculating slope? The figure has 4 significant digits, the text has three.
    - *We have modified all figures and text to include only 3 significant digits.*
- Why is isometric scaling 0.66 - is that microns squared over microns cubed? Please explain.
    - *We have added additional explanation to the results section.*
- Were all four posterior nuclei measured? The figure indicates just M1 and M4.
    - *We apologize that it was not clear that all four posterior nuclei were measured in Fig. 1. For the sake of space, we only showed images of the M1, M4, and Anterior ring canals and nuclei (in Fig. 1A), but all four nuclear measurements were included in the graph in Fig. 1B. We have added M1-M4 to the legend to clarify and revised the text of the legend.*
- It is hard to explain why all four posterior nuclei are bigger than anterior when one of the four is the same age as the anterior nucleus.
    - *The posterior nuclei are larger than the anterior nuclei due to their proximity to the oocyte. Multiple recent studies have described this hierarchical nurse cell size relationship in which the nurse cells closest to the oocyte are larger than those separated from the oocyte by additional intercellular bridges* [3-5].
- In panel D, a conclusion is, "Further, the scaling exponent [slope] for the anterior ring canals, which are also formed during the fourth mitotic division, was not significantly different from that of the posterior M4 ring canals". Anterior is 0.23, M4 is 0.25. These seem different to me. How is significance determined? Were any of the scaling exponents in M1, M2, M3, M4 or Anterior significantly different?
    - *Significance was determined within the Prism software using a method*

© 2024. Published by The Company of Biologists under the terms of the Creative Commons Attribution License (https://creativecommons.org/licenses/by/4.0/).

    - *equivalent to an ANCOVA. If the slopes are compared, M1 is significantly different from M2, M3, and M4, and M2 is significantly different from M4. M4 is not significantly different from the slope for the anterior ring canals, which supports the correlation between scaling and lineage.*
- References are needed for the statements about biased transport to the oocyte.
    - *There was a reference to the Lu (2021) paper in that paragraph, but we have added an additional reference to that paper to this part of the results section.*
- In panel 2C, why are the scaling exponents (slopes) of the controls bigger than in Figure 1B? The controls look hyper allometric in Fig. 2.
    - *This experiment was done with a different GAL4 driver, so it is possible that there are some differences in scaling based on genetic background.*
- In panel 2D it is impossible to pick out the control posterior vs anterior lines - use different colors as in Figure 1. Why do the control lines for posterior and anterior merge?
    - *We have split the ring canal scaling data from Fig. 2D into different separate panels (Fig. 2D,E), as suggested by Reviewer #1.*
    - *These lines likely approach each other because the slope of the line for the anterior ring canals (M4 type) is always larger than the slope for the combined posterior ring canals.*
- Re: Fig. 3: Scaling of what? RC size?
    - *We assume that this comment is related to the heading for this section of the results, so we have added "ring canal to the end of this title, so that it now reads: "Increasing initial ring canal size does not dramatically alter ring canal scaling"*
- Since there was no effect, "dramatically" should be deleted from the section title.
    - *This change has been made.*
- Clarify this sentence: If ring canal size inversely correlates with scaling, then increasing initial ring canal diameter should reduce the scaling exponent.
    - *We have made this change in the text.*
- How does panel B show that RCs are larger in arpC2 KD? Fig. S1A has smaller y- intercept for control. Again, it is impossible to see which lines go with which M and which genotype.
    - *As mentioned above, we have modified Fig. 3 to highlight these differences and added additional explanation to the results section.*
- Panels 4D & 4G are clear - should include significance indications.
    - *We have added asterisks to indicate significant differences.*
- The conclusion from panels 5B and 5C that reducing RC scaling could lead to smaller mature eggs is a stretch. Without looking at the rest of the lines these data are preliminary and detract from the rest of the paper.
    - *As suggested by Reviewer #1, we have modified the results and discussion sections, and we have added a statement about the need for analysis of additional lines.*

4. Description of analyses that authors prefer not to carry out

<u>*Comment from Reviewer #2*</u>

- I am surprised that the author has not considered controlling the impact of cell cycle regulation on this scaling process, especially as the work of Dorherty et al. has shown that this type of regulation is essential for regulating the size of nurse cell nuclei. The authors should test the impact of at least dacapo and cyclin E in this process.
    - *We have attempted to deplete Dacapo from the germline by crossing two different RNAi lines to multiple germline drivers; however, we have been unable to see a consistent effect on nurse cell nuclear size, which suggests that these* RNAi lines may not effectively reduce Dacapo protein in the germline. Although we agree with the reviewer that this is an obvious mechanism that should be explored, we believe that it is not necessary for it to be included in this manuscript, because altering Dacapo levels in the germline would not provide a mechanism to explain our model that ring canal lineage impacts ring canal

© 2024. Published by The Company of Biologists under the terms of the Creative Commons Attribution License (https://creativecommons.org/licenses/by/4.0/).

scaling. Dacapo has been shown to contribute to the hierarchical pattern of nurse cell size observed in the germline. Dacapo mRNA produced in the nurse cells is transported into the oocyte, where it is translated. Then, the Dacapo protein diffuses back into the nurse cells, producing a posterior to anterior gradient [4]. *Doherty (2021) showed that reducing the levels of the Dacapo protein using the deGradFP system eliminated the nurse cell size hierarchy. If our data had supported a model in which proximity to the oocyte was a strong predictor of ring canal size and scaling (as shown for the nurse cells and their nuclei[3,5]), then this would have been an excellent way to dig further into the mechanism. Instead, our data supported a role for ring canal lineage in predicting ring canal growth, since the M4 ring canals at the posterior and anterior showed similar scaling with egg chamber volume.*

- *We believe that performing the proposed experiments (over-expressing HtsRC to increase ring canal size or expressing the phosphomimetic form of the myosin regulatory light chain, $sqh^{E20,E21}$ to reduce ring canal size) will allow us to determine how ring canal size affects scaling, which will provide additional mechanistic insight into this scaling behavior.*

<u>Comment from Reviewer #3</u>

- Panel 3E is interesting and would fit better in Figure 1.
  - *This panel is from a different genetic background than the data in Fig. 1. Therefore, we do not think it would be appropriate to move it to Fig. 1.*

1. Ong S, Tan C. Germline cyst formation and incomplete cytokinesis during *Drosophila melanogaster* oogenesis. *Developmental Biology*. 2010 Jan;337(1):84–98.
2. Gerdes JA, Mannix KM, Hudson AM, Cooley L. HtsRC-Mediated Accumulation of F-Actin Regulates Ring Canal Size During *Drosophila melanogaster* Oogenesis. *Genetics*. 2020 Nov 1;216(3):717–734.
3. Diegmiller R, Doherty CA, Stern T, Alsous JI, Shvartsman SY. Size scaling in collective cell growth. *Development*. 2021 Aug 31;dev.199663.
4. Doherty CA, Diegmiller R, Kapasiawala M, Gavis ER, Shvartsman SY. Coupled oscillators coordinate collective germline growth. *Dev Cell*. 2021 Mar 22;56(6):860-870.e8. PMCID: PMC8265018
5. Imran Alsous J, Villoutreix P, Berezhkovskii AM, Shvartsman SY. Collective Growth in a Small Cell Network. *Current Biology*. 2017 Sep;27(17):2670-2676.e4.

Original submission

<u>First decision letter</u>

MS ID#: DEVELOP/2024/202676

MS TITLE: Lineage-based scaling of germline intercellular bridges during oogenesis

AUTHORS: Umayr Shaikh, Kathleen Sherlock, Julia Wilson, William Gilliland, and Lindsay Lewellyn

I have reviewed the manuscript, the reviews from Review Commons and your plan for revision. I will be happy receive a revised version of the manuscript based on the revision plan provided by you. Your revised paper will be re-reviewed by one or more of the original referees. Please send us a point-by-point response indicating your plans for addressing the referees' comments, and we will look over this and provide further guidance.

Please attend to all of the reviewers' comments and ensure that you clearly highlight all changes made in the revised manuscript. Please avoid using 'Tracked changes' in Word files as these are lost in PDF conversion. I should be grateful if you would also provide a point-by-point response detailing

© 2024. Published by The Company of Biologists under the terms of the Creative Commons Attribution License (https://creativecommons.org/licenses/by/4.0/).

how you have dealt with the points raised by the reviewers in the 'Response to Reviewers' box. If you do not agree with any of their criticisms or suggestions please explain clearly why this is so.

First revision

Author response to reviewers' comments

We would like to thank the reviewers for their time and valuable suggestions. We believe that the changes we have made in response to the reviewer comments have strengthened and streamlined the manuscript.

Responses to Comments from Reviewer #1

- The introductory material and the title of the paper emphasize the ring canal scaling question. This problem is somewhat obscured in the text by the side problem of nuclear scaling, which comes up frequently even though the results are not as thoroughly explored. Could the authors think about moving these data into a different, single figure for the sake of coherence? This is not a required revision. Just a thought.
  - *After making other changes to the text, we agree that with Reviewer #1 that the paper would be stronger by maintaining the focus on the ring canals. Therefore, we have decided to remove most of the nuclear scaling data from this study; we plan to further explore the lineage-based differences in nuclear size and growth in the future.*
- One way to think about the dhc-64C experiments presented in Figure 2 is that they are meant to test the hypothesis that ring canal size impacts scaling in such a way that transport across the four ring canals tends towards equilibrium over time. One possibility would therefore be that ring canals aren't programmed to grow to a particular final size but rather they grow at different rates until their diameters are the same. This seems to me an important distinction. It might be made by analysis of the arpC2-RNAi cells, since those ring canals are meant to be initially larger. Unfortunately, I can't see the answer.
  - *Reviewer #3 suggested determining the ratio of the diameter of the M1 ring canal to the M4 ring canal, which we have included in Fig. 3G. If ring canals grow toward equilibrium (to achieve a similar final size), then we would expect to see this ratio approach 1; when we performed this analysis, we saw that the ratio did decrease as the egg chambers increased in volume, but it never quite reaches a ratio of 1. Interestingly, over-expression of HtsRC::GFP not only increased ring canal diameter but significantly reduced the M1/M4 ratio. This could be because these larger ring canals reach an optimal size or hit an upper limit sooner, or it could be that the structure of these larger ring canals is different. For example, it was previously shown that the ring canals in the HtsRC::GFP over-expressing egg chambers differed in thickness compared to controls (reported to be thicker at earlier stages and thinner at later stages)*(Gerdes et al., 2020). *We did not compare lumen size in our study, but it is possible that this could be used to approximate the amount of material transport through different types of ring canals. Future studies could focus on lumen size differences based on lineage, developmental stage, and across different genetic backgrounds.*
  - *We have added additional discussion of this idea to the results (lines 253-264) and discussion (lines 405-421) sections.*
- The authors write that arpC2-RNAi "ring canals tended to be larger than those in similarly-sized control egg chambers," but that conclusion isn't obvious to me from the data in Figure 3B. The only difference I can see is that the M4 ring canals look to be consistently smaller in the experimental versus control egg chambers, especially at the final timepoint.
  - *Reviewer #3 suggested over-expression of HtsRC::GFP would be a more straightforward way to increase ring canal diameter. We have performed this experiment, and because the HtsRC::GFP over-expression produced a more consistent increase in ring canal size, we chose to replace the* arpC2-RNAi *data*

© 2024. Published by The Company of Biologists under the terms of the Creative Commons Attribution License (https://creativecommons.org/licenses/by/4.0/).

in Figure 3.

- The authors write that "there was a consistent, but not significant decrease in the scaling exponents for the arpC2-RNAi egg chambers compared to controls," but I don't see this in the M1 (identical) or M2 (almost the same) ring canals. The scaling decrease is most pronounced at M4. All the other ring canals seem to reach a final size that's equivalent to controls. What does this tell us about scaling? Is the M4 more sensitive to the effect of arpC2-RNAi? I note and appreciate that the data for M4 show a wide distribution and might have been impacted by outliers, which could be discussed.
    - *We have replaced the* arpC2-RNAi *data in Fig. 3 with date from egg chambers over-expressing HtsRC::GFP. In these egg chambers, we saw a consistent reduction in ring canal scaling compared to control; however, this difference was only significant for the M3 ring canals. We discuss the idea that there could be an upper limit or an optimal size for the ring canals, and that in this condition, the larger ring canals could reach that sooner than the controls (lines 416-421).*
- The possibility that ring canal scaling "could generate eggs of different sizes" could use some elaboration (at least) as it does not seem to be especially well supported:
    - Only one of the small egg lines had lower scaling exponents than the big egg lines, and it's a struggle for me to understand the extent of that difference based on the data shown. (Is it significant?).
        - *We have restructured this section of the results and modified Fig. 5 to highlight similarities and differences between the four lines. In the results section (and in the figure legend), we have stated that when we compared the slopes of the regression lines for all four lines, there was a significant difference for M1, M2, and M4 (Fig. 5C,D,F). We have also modified the results section to highlight that although the slopes for line* 9.31.4 was not different from the two big egg lines, the intercepts were significantly different for M1, M2, M3, and M4 ring canals. We moved the nuclear scaling data to Fig. S2B to simplify the figure.
    - The authors conclude that "the effect of lineage on ring canal scaling is conserved, and it suggests that at least in one line, reducing posterior ring canal scaling could provide a mechanism to produce a smaller mature egg." The first part of this sentence is confusing for me since I don't know what is meant here by "conserved."
        - *We have modified the first part of the sentence to clarify our point that "After completing ring canal measurements, the lineage-based pattern of scaling exponents was consistent with what we had observed previously." (Line 315-316)*
    - The second part of the sentence is technically correct but disguises what I would consider the more meaningful and exciting finding. The 9.31.4 line produces the smallest eggs but does not demonstrate scaling differences in comparison to the big egg lines examined (1.40.1 and 3.34.1). The authors have therefore avoided/solved a "chicken and egg" ("fruit fly and egg"?) problem by showing that scaling and egg size can be decoupled!
        - *We appreciate this suggestion and have modified the text in the results section to further elaborate on differences between the lines (lines 302- 341).*
    - This point is not made very clearly in the discussion, which concludes with the suggestion that scaling could help explain why some insects produce much larger or much smaller eggs that fruit flies. I can only understand this to be the case if - as the authors point out - scaling "affect<s> the directed transfer of materials into the oocyte." That argument seems predicated on the possibility that these insects make the same amount of initial material then regulate how much is transferred. Seems like a costly way to go about it.
        - *We have expanded this part of the discussion and included an example from a recent study on egg size plasticity in honeybees (lines 469-485).*

© 2024. Published by The Company of Biologists under the terms of the Creative Commons Attribution License (https://creativecommons.org/licenses/by/4.0/).

- I really had to look very closely to distinguish the little blue boxes from the little blue circles in panels 2C and especially 2D. I suggest using a different color instead of a different shape, or maybe splitting the graphs up.
  - *We have made the shapes larger in Fig. 2C (posterior ring canal size data), and we have moved the anterior ring canal size data to Fig. S1A.*
- "Depletion of the linker protein, Short stop (Shot), or dynein heavy chain (Dhc64C), significantly reduced the biased transport at the posterior, which reduced oocyte size (Lu et al., 2021)." I suggest this sentence might be clearer if it was rewritten as "Depletion of either dynein heavy chain (Dhc64C) or the linker protein Short stop (Shot) significantly reduces biased transport at the posterior, in turn reducing oocyte size (Lu et al., 2021).
  - *We have made this change (lines 217-219).*
- "Because nuclear growth has been shown to be tightly coupled to cell growth (Diegmiller et al., 2021), we can use nuclear size as a proxy for nurse cell size." I think it would help the reader to know that the Diegmiller study was performed using germline cysts in the Drosophila ovary; I paused when I got to this sentence because I initially read it as overly broad. I suggest "Recent work in demonstrates that nuclear growth is tightly coupled to cell growth in this system (Diegmiller et al., 2021), and we can therefore use nuclear size as a proxy for nurse cell size" or similar. This is certainly not a required revision, just a suggestion.
  - *We have made this change (lines 140-142).*

<u>Responses to Comments from Reviewer #3</u>

- Reviewer #3 asked: Does the ratio of the diameter of M1 to M4 stay the same?
  - *We have performed this analysis in the control and HtsRC::GFP over-expressing egg chambers (Fig. 3G), and we found that the ratio does not stay the same, but that it tends to decrease as the egg chamber increases in volume. Interestingly, HtsRC::GFP over-expression produced larger ring canals, and this ratio was significantly lower than in control egg chambers. Additional work will be necessary to distinguish between the models proposed by Reviewer #1 (whether ring canals grow to a specific target size or whether they grow until transport rates are similar across the four ring canals).*
- It would be helpful to explain that the log-log plots were used to derive a line equation (y=mx + b) and why that is useful in this context. In the case of a log-log plot, what does the y-intercept mean biologically? Is it simply a way to compare two things or does it indicate real measurements such as volume or ring canal size? Also, the slope of the line is being used as a scaling value. Be careful to define the terms "scaling" and "scaling exponent".
  - *We have added additional explanation in the results section (lines 149-159).*
- Are four significant digits called for in calculating slope? The figure has 4 significant digits, the text has three.
  - *We have modified all figures and text to include only 3 significant digits.*
- Why is isometric scaling 0.66 - is that microns squared over microns cubed? Please explain.
  - *Yes, that is correct, and we have added additional explanation to the results section (lines 155-159).*
- **Were all four posterior nuclei measured? The figure indicates just M1 and M4.**
  - *We apologize that it was not clear that all four posterior nuclei were measured in Fig. 1. For the sake of space, we only showed images of the M1, M4, and anterior ring canals and nuclei (in Fig. 1A), but all four nuclear measurements were included in the graph in Fig. 1B. We have added M1-M4 to the legend to clarify and revised the text of the legend.*
- It is hard to explain why all four posterior nuclei are bigger than anterior when one of the four is the same age as the anterior nucleus.
  - *The posterior nuclei are larger than the anterior nuclei due to their proximity to the oocyte. Recent studies have described this hierarchical nurse cell size relationship in which the nurse cells closest to the oocyte are larger than those separated from the oocyte by additional intercellular bridges* (Diegmiller et al., 2021; Doherty et al., 2021; Imran Alsous et al., 2017).

© 2024. Published by The Company of Biologists under the terms of the Creative Commons Attribution License (https://creativecommons.org/licenses/by/4.0/).

- In panel D, a conclusion is, "Further, the scaling exponent [slope] for the anterior ring canals, which are also formed during the fourth mitotic division, was not significantly different from that of the posterior M4 ring canals". Anterior is 0.23, M4 is 0.25. These seem different to me. How is significance determined? Were any of the scaling exponents in M1, M2, M3, M4 or Anterior significantly different?
    - *Significance was determined within the Prism software using a method equivalent to an ANCOVA. If the slopes are compared, M1 is significantly different from M2, M3, and M4, and M2 is significantly different from M4. M4 is not significantly different from the slope for the anterior ring canals, which supports the correlation between scaling and lineage.*
- References are needed for the statements about biased transport to the oocyte.
    - *There was a reference to the Lu (2021) paper in that paragraph, but we have added an additional reference to that paper to this part of the results section (line 219).*
- In panel 2C, why are the scaling exponents (slopes) of the controls bigger than in Figure 1B? The controls look hyper allometric in Fig. 2.
    - *This experiment was done with a different GAL4 driver, so it is possible that there are some differences in scaling based on genetic background.*
- In panel 2D it is impossible to pick out the control posterior vs anterior lines - use different colors as in Figure 1. Why do the control lines for posterior and anterior merge?
    - *We have split the ring canal scaling data into separate panels (Fig. 2C,D,S1A), and we hope that this has made it easier to see the data points.*
    - *These lines likely approach each other because the slope of the line for the anterior ring canals (M4 type) was larger than the slope for the combined posterior ring canals.*
- I cannot see differences in RC size in the panel A images. More importantly, this method altering ring canal size is limited. A more direct way is overexpression of HtsRC (https://doi.org/10.1534/genetics.120.303629).
    - *We appreciate this suggestion, and we have performed the suggested experiment, which is now included in Fig. 3.*
    - *We did try to determine the effect of reducing ring canal size by expressing a phosphomimetic version of the non-muscle myosin II regulatory light chain, encoded by spaghetti squash (Sqh)(UAS-sqh^{E20E21}), in the germline. Although this condition did reduce the size of the nurse cell-nurse cell ring canals, we did not see a consistent reduction in the size of the four posterior nurse cell-oocyte ring canals. We did anticipate this problem, since a study that looked at egg chambers homozygous for a mutation in the myosin binding subunit of the myosin phosphatase, DMYPT, which should also increase sqh phosphorylation, found that the posterior ring canals were larger than those connecting nurse cells* (Ong and Tan, 2010). *We have added a point in the discussion (lines 433-440) that we would like to perform additional studies on other manipulations that would increase or decrease ring canal size.*
- In panel 2E, it would be helpful to plot the y-intercepts separately, too.
    - *We have plotted the y-intercepts in Fig. S1B.*
- Re: Fig. 3: Scaling of what? RC size?
    - *We assume that this comment is related to the heading for this section of the results, so we have added "ring canal", so that it now reads: "Increasing ring canal size does not affect the lineage-based patterns, but it does lead to modest differences in scaling" (lines 240-241).*
- **Since there was no effect, "dramatically" should be deleted from the section title.**
    - *This was removed when we modified the figure.*
- Clarify this sentence: If ring canal size inversely correlates with scaling, then increasing initial ring canal diameter should reduce the scaling exponent.
    - *We have made this change in the text (lines 242-244). It now reads: "*If ring canal size inversely correlates with scaling such that larger ring canals increase in size more slowly than smaller ring canals, then increasing ring canal diameter should reduce the scaling exponent."
- How does panel B show that RCs are larger in arpC2 KD? Fig. S1A has smaller y- intercept

© 2024. Published by The Company of Biologists under the terms of the Creative Commons Attribution License (https://creativecommons.org/licenses/by/4.0/).

for control. Again, it is impossible to see which lines go with which M and which genotype.
  - *We have modified Fig. 3 to focus on the over-expression of HtsRC::GFP.*
- Panel 3E is interesting and would fit better in Figure 1.
  - *This panel was from a different genetic background than the data in Fig. 1, and since we have now modified Fig. 3 to focus on the HtsRC::GFP over-expression data, we did not make this change.*
- Panels 4D & 4G are clear - should include significance indications.
  - *We have added asterisks to indicate significant differences.*
- The conclusion from panels 5B and 5C that reducing RC scaling could lead to smaller mature eggs is a stretch. Without looking at the rest of the lines these data are preliminary and detract from the rest of the paper.
  - *As suggested by Reviewer #1, we have modified the results and discussion sections, and we have added a statement about the need for analysis of additional lines or additional species that produce much larger or much smaller mature eggs.*

<u>Response to Comment from Reviewer #2</u>

- I am surprised that the author has not considered controlling the impact of cell cycle regulation on this scaling process, especially as the work of Dorherty et al. has shown that this type of regulation is essential for regulating the size of nurse cell nuclei. The authors should test the impact of at least dacapo and cyclin E in this process.
  - *We have attempted to deplete Dacapo from the germline by crossing two different RNAi lines to multiple germline drivers; however, we have been unable to see a consistent effect on nurse cell nuclear size, which suggests that these RNAi lines may not effectively reduce Dacapo protein in the germline. Although we agree with the reviewer that this is an obvious mechanism that should be explored, we believe that it is not necessary for it to be included in this manuscript, because altering Dacapo levels in the germline would not provide a mechanism to explain our conclusion that ring canal lineage impacts ring canal scaling. Dacapo has been shown to contribute to the hierarchical pattern of nurse cell size observed in the germline. Dacapo mRNA produced in the nurse cells is transported into the oocyte, where it is translated. Then, the Dacapo protein diffuses back into the nurse cells, producing a posterior to anterior gradient* (Doherty et al., 2021). *Doherty (2021) showed that reducing the levels of the Dacapo protein using the deGradFP system eliminated the nurse cell size hierarchy. If our data had supported a model in which proximity to the oocyte was a strong predictor of ring canal size and scaling (as shown for the nurse cells and their nuclei* (Diegmiller et al., 2021; Imran Alsous et al., 2017)*), then this would have been an excellent way to dig further into the mechanism. Instead, our data* supported a role for ring canal lineage in predicting ring canal growth, since the M4 ring canals at the posterior and anterior showed similar scaling with egg chamber volume.

Diegmiller, R., Doherty, C. A., Stern, T., Alsous, J. I. and Shvartsman, S. Y. (2021). Size scaling in collective cell growth. *Development* dev.199663.

Doherty, C. A., Diegmiller, R., Kapasiawala, M., Gavis, E. R. and Shvartsman, S. Y. (2021). Coupled oscillators coordinate collective germline growth. *Dev Cell* 56, 860-870.e8.

Gerdes, J. A., Mannix, K. M., Hudson, A. M. and Cooley, L. (2020). HtsRC-Mediated Accumulation of F-Actin Regulates Ring Canal Size During Drosophila melanogaster Oogenesis. *Genetics* **216**, 717–734.

Imran Alsous, J., Villoutreix, P., Berezhkovskii, A. M. and Shvartsman, S. Y. (2017). Collective Growth in a Small Cell Network. *Current Biology* **27**, 2670-2676.e4.

Ong, S. and Tan, C. (2010). Germline cyst formation and incomplete cytokinesis during Drosophila melanogaster oogenesis. *Developmental Biology* **337**, 84–98.

<u>Second decision letter</u>

© 2024. Published by The Company of Biologists under the terms of the Creative Commons Attribution License (https://creativecommons.org/licenses/by/4.0/).

MS ID#: DEVELOP/2024/202676

MS TITLE: Lineage-based scaling of germline intercellular bridges during oogenesis

AUTHORS: Umayr Shaikh, Kathleen Sherlock, Julia Wilson, William Gilliland, and Lindsay Lewellyn

I have now received all the referees reports on the above manuscript, and have reached a decision. The referees' comments are appended below, or you can access them online: please go to BenchPress and click on the 'Manuscripts with Decisions' queue in the Author Area.

The overall evaluation is positive and we would like to publish a revised manuscript in Development. However, the reviewers provide suggestions to further improve the clarity and rigor in the analysis. While I do not expect to send the manuscript back to the reviewers, I will look at it carefully, so please attend to all of the reviewers' comments in your revised manuscript and detail them in your point-by-point response. If you do not agree with any of their criticisms or suggestions explain clearly why this is so.

Reviewer 1

Advance summary and potential significance to field

In this manuscript Umayr Shaikh and colleagues investigate how size of subcellular structures is evolving during tissue growth. To do so, the authors are using the drosophila egg chamber as model system by investigating in the germline cyst the growth rate of the intercellular bridges also named ring canals connecting the oocyte to the nurse cells during egg chamber development. They show that ring canal size scaling is related to lineage. Analysis of a set of artificially selected “big egg” and “small egg” lines revealed that the lineage dependent pattern of scaling is maintained, but that subtle changes to ring canal scaling could provide a mechanism to regulate final egg size.

Comments for the author

In this revised manuscript, Umayr Shaikh and colleagues have made several clarifications that improve their manuscript and modified the figures accordingly.
The authors gave adequate answers to the points I raised, although they were unable to analyse Dacapo’s involvement. They provided enough detailed information to the comments and questions submitted by the reviewers.
The manuscript has improved since the last submission and I would recommend its publication in Development.

Reviewer 2

Advance summary and potential significance to field

Ring canals, intercellular bridges, which originate from an arrested cleavage furrow at the end of cell division, connect the 16 cells within a germline cyst in Drosophila follicles and allow transport of molecules from nurse cells to the oocyte. Growth of ring canals coincides with the growth of nurse cells and the whole egg chamber. Here, the authors use the ring canals as a model for investigating the scaling of subcellular structures during development.

The authors investigated the scaling of ring canals in relation to their location in the egg chamber (anterior vs. posterior) and time of formation (lineage). (1) Results from wild-type analysis indicate that the biggest ring canal, which is derived from the first mitotic division, grows slower than the smaller ring canals derived from the last (4th) mitotic division independent of their position in the egg chamber and growth pattern of nurse cells. The authors conclude that the scaling of ring canals depends on their lineage. (2) The authors use various genetic manipulations that alter transport through ring canals, size (increase or decrease) or number of ring canals, or final egg size to determine whether any of those parameters might influence the scaling of ring canals. Although

© 2024. Published by The Company of Biologists under the terms of the Creative Commons Attribution License (https://creativecommons.org/licenses/by/4.0/).

slight effects were quantified on ring canal growth when ring canals were generally bigger or smaller, scaling remained largely dependent on lineage in all conditions tested.

Although the authors did not identify a molecular mechanism that controls the different scaling of ring canals, their data convincingly point to ring canal lineage as the underlying cause for differences in scaling. As such, this study serves as an important guide for future projects that aim to find the mechanism behind this scaling phenomenon.

Comments for the author

The manuscript is written in a transparent and clear manner and is properly edited. Data are demonstrated in clear images and graphs, and are well supported by the careful quantitative analysis.

A. Critique points

1. I am wondering how accurate the ring canal measurements are considering that images were used that were taken with a low magnification (20X) objective. Further, it was not explained how these measurements were conducted (see point B3). I like to suggest that the authors test the accuracy of their measurements by repeating some of the core measurements in wild-type using images taken with a 40 X objective.

2. Sample size:

The authors state in legends for figures 2-5 that two experiments were conducted but results only show data from one experiment. Does the sample sizes refer to the experiment that is illustrated in the graphs, or to both experiments together. It is important to clarify this for Figures 3-5.

Was the second experiment evaluated? Are the data from the two experiments consistent? In my opinion, to point out that a second experiment was done if the data are not included does not add anything to the manuscript. I suggest to either include all experiments (showing the data from the second experiment in graphs or tables in supplementary materials) or to delete statements about a 2nd experiment.

B. Minor points:

2. Abstract and Intro:

- Replace 'germ cells' with 'germline cells' (line 24, 25, 30, 92, and throughout manuscript)

- line 78/79: change to: After completion of the four mitotic divisions, the ring canals .....

- line 89: consider changing to: ....compared to smaller, later born ring canals .....

- line 84 and 139: change to: ......anterior most nurse cell ..... (posterior most nurse cell)

3. Material and Methods:

- Flystocks: please, list the big and small egg lines

- Ring canal ... measurements: Please, describe in more detail how the diameter of ring canals was measured as images often show ring canals in a tilted position or as a tangential cut. Same for measurements of nuclei. Please, add a description of the volume measurement for egg chambers (only mature eggs are described).

- Statistics: Please, add a chapter, summarizing the various statistical methods that were used.

4. Results:

- line 273: change to: ..construct was used to induce a depletion of .....

© 2024. Published by The Company of Biologists under the terms of the Creative Commons Attribution License (https://creativecommons.org/licenses/by/4.0/).

5. Figures:

- explain abbreviations, such as RC and EC in main figures/figure legends and Supplementary figure legends

- Figure 1A legend: indicate oogenesis stages for panel (A)

- Figure 1A legend: add 50 µm scale bar description for panel (A). (Also it is shown in the figure, it is too small to be readable). Please, add this also to legends of Figures 2-4.

- Figure 1A: Authors use nice color-coding, but I would suggest to also add the identifier 'anterior', 'M4' and 'M1' to the Figure panels to make it easier for the reader

---

Second revision

<u>Author response to reviewers' comments</u>

<u>Response to Reviewer #2 Comments to Author</u>

A. Critique points

1. I am wondering how accurate the ring canal measurements are considering that images were used that were taken with a low magnification (20X) objective. Further, it was not explained how these measurements were conducted (see point B3). I like to suggest that the authors test the accuracy of their measurements by repeating some of the core measurements in wild-type using images taken with a 40X objective.

The 20x objective was chosen to allow us to capture the entire egg chamber at later stages of oogenesis. With higher magnification, the field of view was not large enough to fit stage 10 (and sometimes stage 9) egg chambers. To test the accuracy of our measurements, we have done one round of analysis to compare the measurements of the posterior ring canals with both a 20x objective and a 40x objective in control egg chambers (w^{1118}*;;mat□Tub-GAL4; n=72 egg chambers). When we compared the measurements made using the two objectives, we did have an average of 2.85% difference. However, those differences did not lead to any changes in the lineage-based pattern of ring canal scaling, and there were no significant differences in the slopes or y-intercepts of the regression lines (see below).*

NOTE: We have removed unpublished data that had been provided for the referees in confidence.

2. Sample size:

The authors state in legends for figures 2-5 that two experiments were conducted but results only show data from one experiment. Does the sample sizes refer to the experiment that is illustrated in the graphs, or to both experiments together. It is important to clarify this for Figures 3-5. Was the second experiment evaluated? Are the data from the two experiments consistent? In my opinion, to point out that a second experiment was done if the data are not included does not add anything to the manuscript. I suggest to either include all experiments (showing the data from the second experiment in graphs or tables in supplementary materials) or to delete statements about a 2nd experiment.

The samples sizes in the legends did refer to the number of egg chambers analyzed for a single experiment (which was shown in each of the figures). We have removed the statement about the second experiment from each of the legends.

© 2024. Published by The Company of Biologists under the terms of the Creative Commons Attribution License (https://creativecommons.org/licenses/by/4.0/).

B. Minor points:

2. Abstract and Intro:

- Replace 'germ cells' with 'germline cells' (line 24, 25, 30, 92, and throughout manuscript)

This change has been made.

- line 78/79: change to: After completion of the four mitotic divisions, the ring canals .....

This change has been made.

- line 89: consider changing to: ....compared to smaller, later born ring canals .....

This change has been made.

- line 84 and 139: change to: ......anterior most nurse cell (posterior most nurse cell)

This change has been made.

3. Material and Methods:

- Flystocks: please, list the big and small egg lines

The stocks have been added.

- Ring canal ...... measurements: Please, describe in more detail how the diameter of ring canals was measured as images often show ring canals in a tilted position or as a tangential cut. Same for measurements of nuclei. Please, add a description of the volume measurement for egg chambers (only mature eggs are described).

This has been added.

- Statistics: Please, add a chapter, summarizing the various statistical methods that were used.

This has been added.

4. Results:

- line 273: change to: ..construct was used to induce a depletion of .....

This change has been made.

5. Figures:

- explain abbreviations, such as RC and EC in main figures/figure legends and Supplementary figure legends

This has been added.

- Figure 1A legend: indicate oogenesis stages for panel (A)

This has been added.

- Figure 1A legend: add 50 μm scale bar description for panel (A). (Also it is shown in the figure, it is too small to be readable). Please, add this also to legends of Figures 2-

© 2024. Published by The Company of Biologists under the terms of the Creative Commons Attribution License (https://creativecommons.org/licenses/by/4.0/).

4.

This has been added.

- Figure 1A: Authors use nice color-coding, but I would suggest to also add the identifier 'anterior', 'M4' and 'M1' to the Figure panels to make it easier for the reader

This has been added.

Third decision letter

MS ID#: DEVELOP/2024/202676

MS TITLE: Lineage-based scaling of germline intercellular bridges during oogenesis

AUTHORS: Umayr Shaikh, Kathleen Sherlock, Julia Wilson, William Gilliland, and Lindsay Lewellyn

Thank you for sending your manuscript to Development through Review Commons

I am happy to tell you that your manuscript has been accepted for publication in Development, pending our standard publication integrity checks.

© 2024. Published by The Company of Biologists under the terms of the Creative Commons Attribution License (https://creativecommons.org/licenses/by/4.0/).
